# Lymphatic pathway evaluation in congenital heart disease using 3D whole-heart balanced steady state free precession and T2-weighted cardiovascular magnetic resonance

**DOI:** 10.1186/s12968-021-00707-6

**Published:** 2021-03-01

**Authors:** Vasu D. Gooty, Surendranath R. Veeram Reddy, Joshua S. Greer, Zachary Blair, Riad Abou Zahr, Yousef Arar, Daniel A. Castellanos, Sheena Pimplawar, Gerald F. Greil, Jeanne Dillenbeck, Tarique Hussain

**Affiliations:** 1 Department of Pediatrics, Division of Pediatric Cardiology, University of Tennessee Health Science Center, Le Bonheur Children’s Hospital, 49N Dunlap Street, 3rd Floor, Memphis, TN 38015 USA; 2 Department of Pediatrics, Division of Pediatric Cardiology, University of Texas Southwestern Medical Center, Dallas Children’s Medical Center, Dallas, TX USA; 3 University of Texas Southwestern Medical School, Dallas, TX USA; 4 Department of Pediatric Radiology, University of Texas Southwestern Medical Center, Dallas Children’s Medical Center, Dallas, TX USA

**Keywords:** Congenital heart disease, Interventional CMR, Cardiac catheterization, Magnetic resonance imaging, Lymphatic intervention, T2-weighted imaging, 3D-balanced SSFP, SSFP, Lymphatic imaging, Single ventricle

## Abstract

**Background:**

Due to passive blood flow in palliated single ventricle, central venous pressure increases chronically, ultimately impeding lymphatic drainage. Early visualization and treatment of these malformations is essential to reduce morbidity and mortality. Cardiovascular magnetic resonance (CMR) T2-weighted lymphangiography (T2w) is used for lymphatic assessment, but its low signal-to-noise ratio may result in incomplete visualization of thoracic duct pathway. 3D-balanced steady state free precession (3D-bSSFP) is commonly used to assess congenital cardiac disease anatomy. Here, we aimed to improve diagnostic imaging of thoracic duct pathway using 3D-bSSFP.

**Methods:**

Patients underwent CMR during single ventricle or central lymphatic system assessment using T2w and 3D-bSSFP. T2w parameters included 3D-turbo spin echo (TSE), TE/TR = 600/2500 ms, resolution = 1 × 1 × 1.8 mm, respiratory triggering with bellows. 3D-bSSFP parameters included electrocardiogram triggering and diaphragm navigator, 1.6 mm isotropic resolution, TE/TR = 1.8/3.6 ms. Thoracic duct was identified independently in T2w and 3D-bSSFP images, tracked completely from cisterna chyli to its drainage site, and classified based on severity of lymphatic abnormalities.

**Results:**

Forty-eight patients underwent CMR, 46 of whom were included in the study. Forty-five had congenital heart disease with single ventricle physiology. Median age at CMR was 4.3 year (range 0.9–35.1 year, IQR 2.4 year), and median weight was 14.4 kg (range, 7.9–112.9 kg, IQR 5.2 kg). Single ventricle with right dominant ventricle was noted in 31 patients. Thirty-eight patients (84%) were status post bidirectional Glenn and 7 (16%) were status post Fontan anastomosis. Thoracic duct visualization was achieved in 45 patients by T2w and 3D-bSSFP. Complete tracking to drainage site was attained in 11 patients (24%) by T2w vs 25 (54%) by 3D-bSSFP and in 28 (61%) by both. Classification of lymphatics was performed in 31 patients.

**Conclusion:**

Thoracic duct pathway can be visualized by 3D-bSSFP combined with T2w lymphangiography. Cardiac triggering and respiratory navigation likely help retain lymphatic signal in the retrocardiac area by 3D-bSSFP. Visualizing lymphatic system leaks is challenging on 3D-bSSFP images alone, but 3D-bSSFP offers good visualization of duct anatomy and landmark structures to help plan interventions. Together, these sequences can define abnormal lymphatic pathway following single ventricle palliative surgery, thus guiding lymphatic interventional procedures.

## Introduction

Patients with single ventricle physiology present unique challenges and require close surveillance after palliative surgical procedures such as superior cavopulmonary anastomosis (Glenn anastomosis) and total cavopulmonary anastomosis (Fontan palliation) [1]. Passive flow in the Fontan conduit and decreased cardiac output lead to increased central venous pressure and chronic venous stasis, eventually causing impeded lymphatic flow [2–5] and lymphatic complications. Thus, early, accurate assessment of the complete lymphatic pathway is critical in reducing overall morbidity and mortality.

Lymphatic imaging by cardiovascular magnetic resonance (CMR) involves T2-weighted (T2w) lymphangiography, which targets high water content structures with long T2 values [6, 7], and dynamic contrast cardiovascular magnetic resonance lymphangiography (DCMRL) [8, 9]. T2w imaging provides excellent anatomical delineation since most tissues are suppressed, but its low signal-to-noise ratio (SNR) occasionally leads to incomplete tracking of the thoracic duct.

3D whole-heart imaging using balanced steady-state free precession (3D-bSSFP) has revolutionized congenital heart disease assessment [10]. 3D-bSSFP images are T2w/T1-weighted, providing desirable contrast for bright blood imaging, and are often acquired in patients requiring lymphatic evaluation. Lymphatic fluids also appear bright in 3D-bSSFP whole-heart acquisitions, providing a complimentary dataset of lymphatic anatomy. Here, we aimed to improve diagnostic visualization of the thoracic duct pathway from cisterna chyli to its drainage site by using 3D-bSSFP combined with T2w to guide interventional lymphatic procedures.

## Methods

We conducted a retrospective review of patients who underwent clinically indicated comprehensive CMR studies as a part of pre-Fontan, pre-Glenn evaluation or for central lymphatic system evaluation (where clinically indicated) with and without invasive CMR from November 2017 to the end of August 2019. CMR was performed on an 1.5T CMR scanner (Ingenia, Philips Healthcare, Best, The Netherlands) with a 32-channel phased array digital receiver torso coil at Dallas Children’s Medical Center and the University of Texas Southwestern Medical Center, Dallas. All studies were performed under general anesthesia.

### T2-weighted lymphangiography

T2w lymphangiography was performed using coronal segmented 3D turbo spin echo (TSE) acquisition with respiratory triggering performed using respiratory bellows at end expiration, a field-of-view (FOV) of 280–400 mm, TE/TR = 700/2500 ms, echo train length = 145, shot duration = 712 ms, refocusing flip angle = 140°, and resolution of 1 × 1 × 1.8 mm. The scan time was 2–5 min, depending on the respiratory rate and patient size. All patients underwent T2w imaging prior to gadolinium-based contrast administration.

### 3D balanced-steady state free precession

3D whole-heart bSSFP images were acquired with a FOV of 280–400 mm (coronal orientation), 1.6–1.8 mm isotropic resolution, TE/TR = 1.8/3.6 ms, SENSE factor of 1.5 in both phase and slice directions, and a flip angle of 60°. Fat suppression and T2-preparation pulses were used for improved visualization of the vasculature [11]. The acquisition was cardiac-triggered to end-systole or mid-diastole, and a diaphragmatic respiratory navigator with a 0.6 tracking factor and 5-mm window was used to reduce respiratory motion artifacts.

### Analysis

T2w and 3D-bSSFP datasets were analyzed on two independent occasions by an initial reader (VG) to identify and track the thoracic duct from cisterna chyli to its drainage site. A second reader (YA) blinded to the results of first reader reviewed all images to identify and track the thoracic duct in both T2w and 3D-bSSFP sequences. Intra- and inter-rater reliability were evaluated using Cohen’s Kappa (κ) statistic. Disagreements between readers were resolved by adjudication by a third reader (TH) with more than 10 years of CMR imaging experience.

Incomplete visualization of the thoracic duct was defined as image blurring to an extent that the observer could not be diagnostically certain that the vessel was intact.

Lymphatic abnormalities were identified and classified by malformation severity. This classification was independently performed based on abnormal lymphatic channel hyperintensity by T2w imaging within the neck and chest [12, 13]. Abnormal lymphatics were classified as follows: type 1: minimal amount of lymphatic channels in the mediastinum or neck; type 2: increased signal intensity within bilateral supraclavicular regions without extension into the mediastinum; type 3: extension of lymphatic channels into the mediastinum; and type 4: extensive involvement of supraclavicular, mediastinum and interstitial pattern in the lung parenchyma.

This study was approved by the University of Texas Southwestern Medical Center Institutional Review Board (IRB Number STU-032016009).

## Results

Forty-eight patients who underwent comprehensive CMR during the study period were screened during single ventricle evaluation and for suspected lymphatic malformations.

**Fig. 1:**
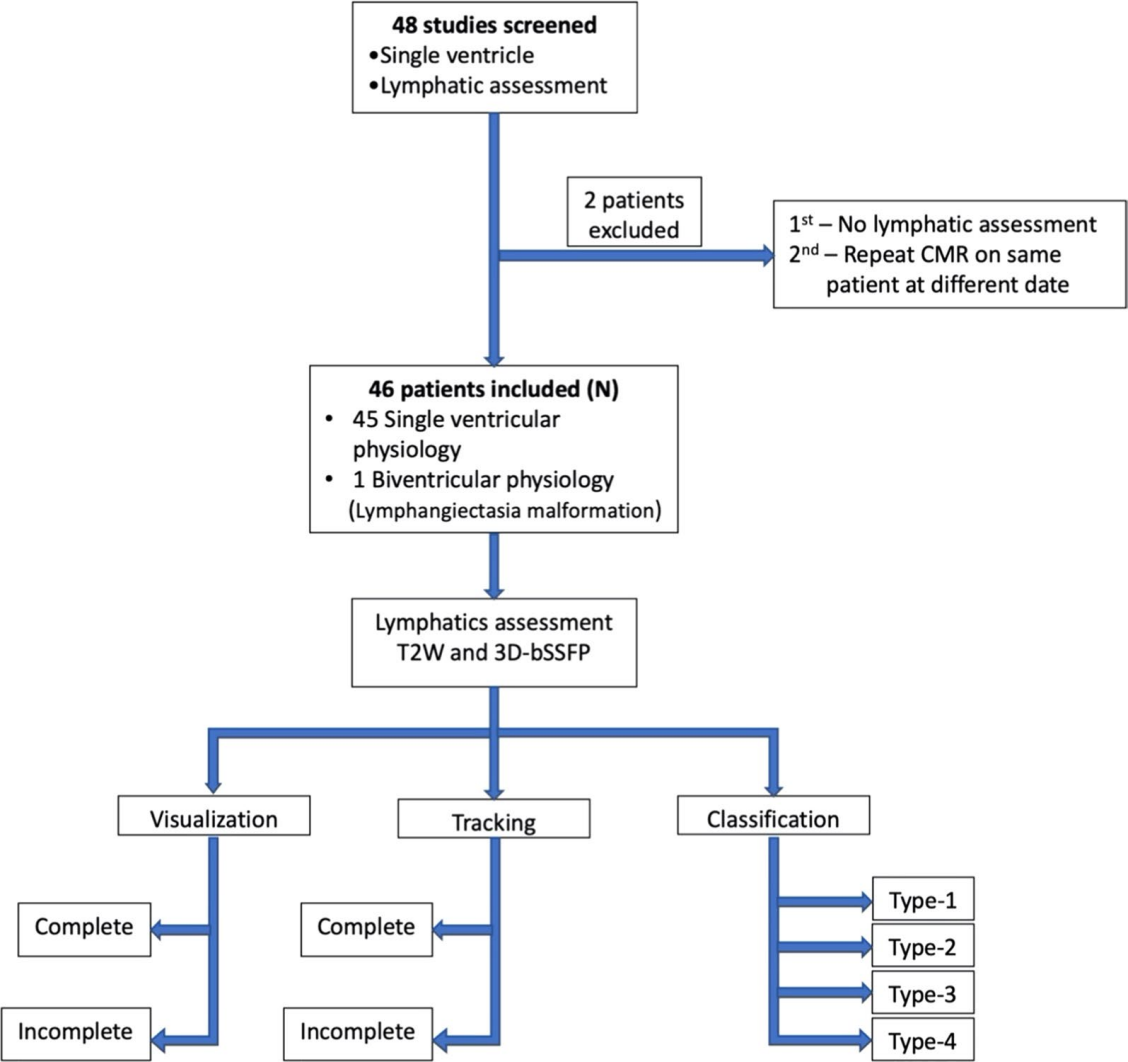
Flowsheet showing the screening, inclusion and assessment of patients

Two studies were excluded; one patient did not undergo lymphatic evaluation during single ventricle imaging, and another patient underwent repeat CMR on a different date. Forty-six patients were included in the study cohort (Fig. 1). Of those, 45 patients had single ventricle physiology, and 1 patient had biventricular physiology but otherwise normal intracardiac anatomy and was referred for lymphangiectasia malformation assessment. Table 1 shows the clinical characteristics of the cohort. The median age of the cohort at CMR was 4.3 year (range, 0.9–35.1 year, IQR 2.4 year), and median weight was 14.4 kg (range, 7.9–112.9 kg, IQR 5.2 kg). Thirty-one patients with single ventricle had right dominant (morphological) ventricle. Most patients had undergone bidirectional Glenn anastomosis (38/45, 84%), followed by Fontan anastomosis (7/45, 16%).

**Table 1:** Clinical charecteristics of the cohort

Demographic characteristics (N = 46)	
Median age (years)	4.3 (0.9–35.1; IQR 2.4)
Median height (cm)	101.3 (70–185; IQR 13.8)
Median weight (kg)	14.4 (7.9–112.9; IQR 5.2)
Median BSA (m^2^)	0.67 (0.4–2.4; IQR 0.13)
Male: Female	33:13

Single ventricle characteristics (45 patients)		
	RV dominant ventricle (69%)	LV dominant ventricle (31%)
Bidirectional Glenn anastomosis (n = 38)	29	9
Fontan anastomosis (n = 7)	2	5

*BSA* Body surface area

All 46 patients underwent lymphatic assessment; we identified and visualized the thoracic duct using both T2w and 3D-bSSFP in 45 patients (~ 98%) (Table 2). Complete tracking of the thoracic duct pathway from cisterna chyli to its drainage site was achieved by T2w imaging in 11 patients (24%), 3 of whom were exclusively identified on T2w. Complete tracking was achieved by 3D-bSSFP in 25 patients (54%), 17 of whom were tracked exclusively using 3D-bSFFP*.* With the combined results of T2w and 3D-bSSFP, complete tracking was possible in 28 patients (61%). T2w and 3D-bSSFP also showed excellent intra-observer reliability (κ = 0.82 and 0.91, respectively) and inter-observer reliability (κ = 0.87 and 0.82, respectively).

**Table 2:** Lymphatic charecteristics of the cohort

	T2-weighted lymphangiography	3D-bSSFP whole heart
Lymphatic characteristics (46 patients)		
Visualization of lymphatics	45 (97.8%)	45 (97.8%)
Complete pathway tracking	11 (24%)	25 (54%)
Lymphatic abnormality classification (31 patients)		
Type 1	7 (23%)	
Type 2	14 (45%)	
Type 3	7 (23%)	
Type 4	3 (9%)	

We graded lymphatic leakage mainly on T2w imaging. Severe lymphatic leaks were also seen on 3D-bSSFP. In this case, 3D-bSSFP complemented T2w imaging and confirmed significant leakage.

**Fig. 2:**
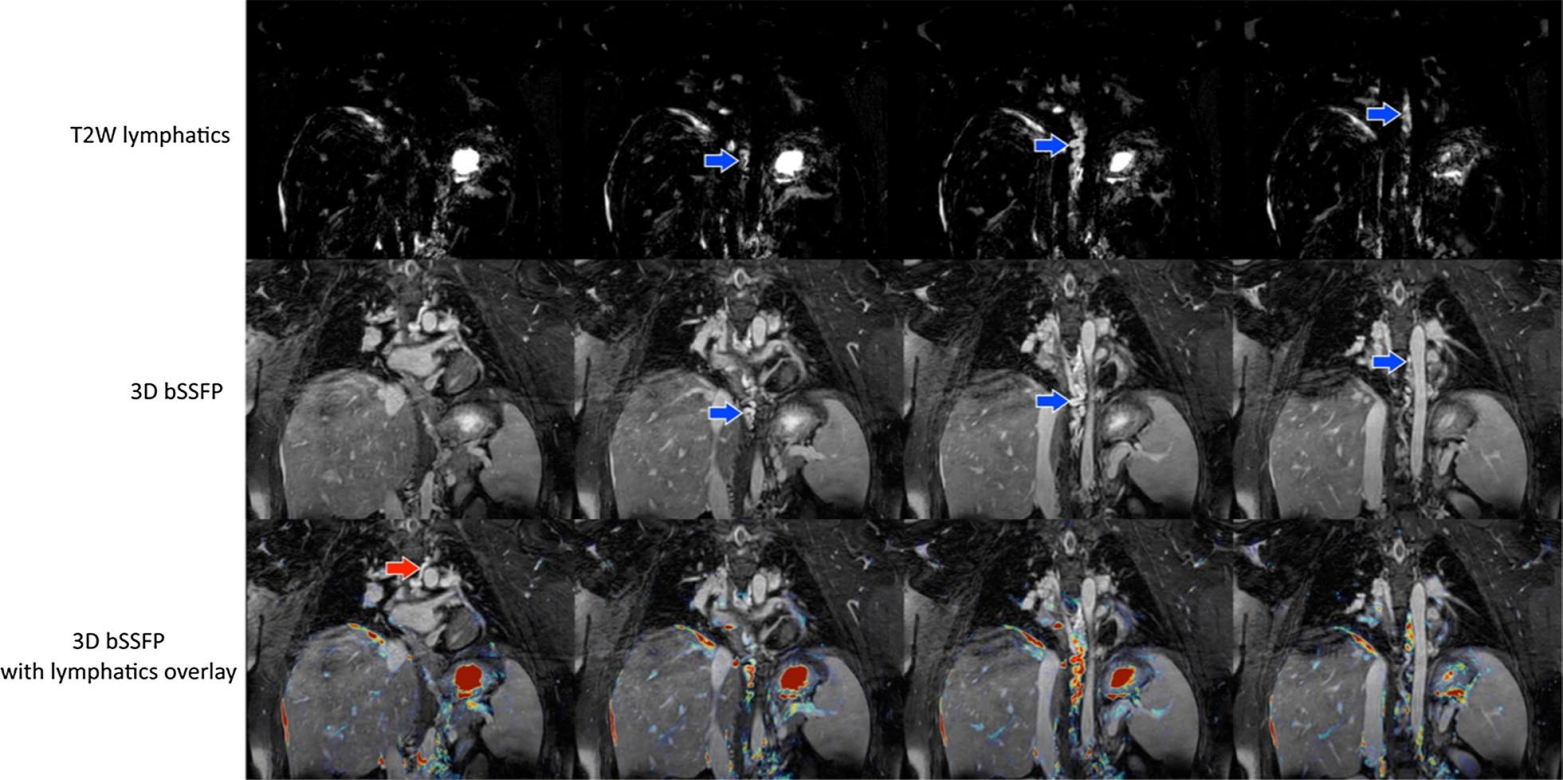
Four slices through the thoracic duct (blue arrows) shown with the T2 weighted (T2w) scan, 3D-balanced steady state free precession (bSSFP), and overlay used to identify lymphatic pathway on 3D-bSSFP. The red arrow indicates a portion of the thoracic duct that was not visualized with the T2w scan but could be seen with 3D-bSSFP (corresponding Additional file 1)

To improve the overall confidence in tracking the thoracic duct pathway, we overlaid two co-registered scans (3D-bSSFP and T2w) in select cases or where intervention was necessary (Fig. 2 and Additional file 1). We classified abnormal lymphatics in 31 patients (Table 2) for whom we had complete tracking of the thoracic duct. Type 2 lymphatic abnormality was the most common finding (14/31, 45%), while type 4 was rare (3/31, 9%). DCMRL was performed in 4 patients; the complete thoracic duct pathway was tracked consistently in 3 patients by 3D-bSSFP vs 1 by T2w imaging.

## Discussion

Here, we demonstrate the complementary role of 3D-bSSFP to T2w lymphangiography in defining the complete lymphatic pathway and associated malformations. Using 3D-bSSFP and T2w markedly improved our overall imaging confidence in the detection and tracking of abnormal lymphatic drainage site(s) and leaks as well as the classification of malformations. Complete tracking of the thoracic duct from cisterna chyli to its drainage site was achieved in 54% of patients by 3D-bSSFP, 24% by T2w imaging, and 61% by both techniques. This complementary imaging guided successful catheter-based lymphatic interventions.

Lymphatic abnormalities are increasingly recognized in single ventricle patients who have undergone palliative surgeries such as bidirectional Glenn anastomosis and the Fontan procedure. Patients with congenital heart disease have abnormal lymphatic findings such as dilation and tortuosity of the thoracic duct, lymphangiectasia, lymphatic collateralization and lymphatic leaks [14, 15]. Chronic venous stasis secondary to increased central venous pressure, lymphatic pressure, impaired central lymphatic drainage mechanisms, and increased lymphatic production [3–5, 16, 17] cause lymph leakage into lower pressure cavities such as the bronchial airway tree. This leakage can drive complications such as plastic bronchitis and protein-losing enteropathy [18–20], ultimately contributing to overall morbidity and mortality [16, 19, 21–25].

Here, we utilized a 3D-bSSFP sequence to assess the lymphatic pathway and its drainage. Since 3D-bSSFP provides T2/T1 contrast and high SNR [26–28], fluid (blood, lymph, cerebrospinal fluid) appears bright. This property allowed identification and complete tracking of the central lymphatic system from cisterna chyli through the thoracic duct to its drainage site in the thorax. We also classified abnormalities by complementing 3D-bSSFP with T2w images, achieving overall visualization of the thoracic duct on par with that of T2w (~ 98%).

Due to the bright fluid signal in 3D-bSSFP images, pleural effusion and complex collaterals can make delineating the thoracic duct track and assessing the precise site of leakage challenging. Thus, we performed CMR angiography in all the patients who underwent lymphatic assessment and 3D-bSSFP. We used multiplanar reconstruction for 3D-bSSFP and CMR angiography, and the reviewing authors (VG, TH) carefully performed a visual correlation to confirm true thoracic duct vs abnormal collaterals. Additionally, by combining and overlaying the thoracic duct with T2w lymphangiography, we obtained complete pathway identification on 3D-bSSFP datasets and identified the lymphatic leak site in selected severe cases.

We were able to visually track complete thoracic duct pathway more often with 3D-bSSFP (54%) than with T2w scans (24%). Incomplete visualization in T2w images was attributed to (1) poor or incomplete tracking of T2w signal and (2) loss of signal in the retrocardiac region. The most common sites of signal loss were in the mid-thorax region where the thoracic duct crosses below the carina and prior to entering the supraclavicular region. This signal loss may have been in part due to cardiac or respiratory motion during the long TSE readout and low SNR and spatial resolution offered by the heavily T2w sequence. Cardiac-triggered scans with a TSE readout optimized for reduced motion sensitivity may be used to minimize retrocardiac signal loss [29].

**Fig. 3:**
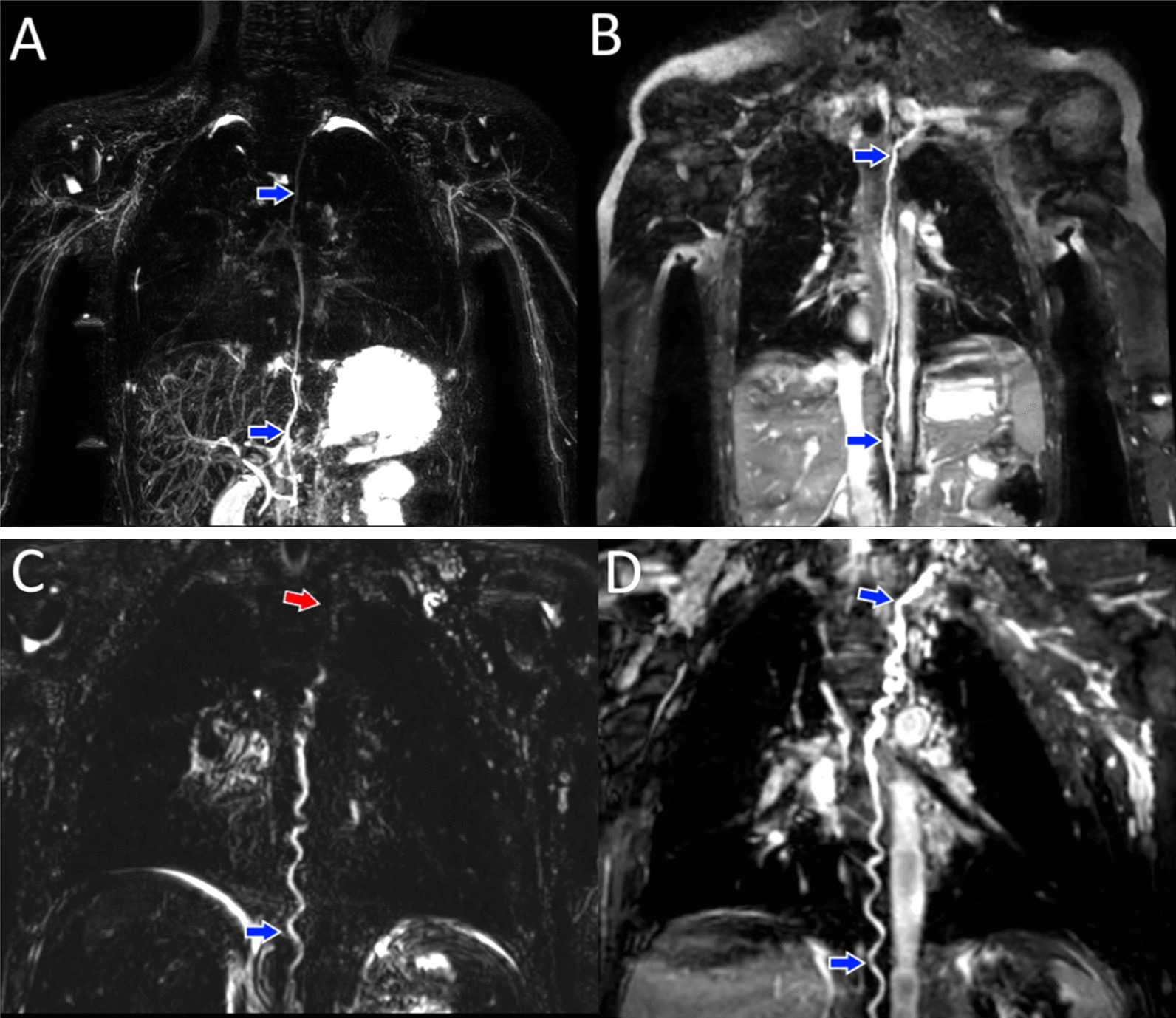
**a** Maximum intensity projection (MIP) of a T2w scan with the entire thoracic duct (blue arrows) visualized. **b** Curved planar reformat of the same patient (**a**) showing the thoracic duct with 3D-bSSFP, also visualized in its entirety. **c** MIP of a T2w lymphatic scan in another patient, with loss of thoracic duct visualization in the upper thorax (red arrow), while (**d**) 3D-bSSFP showed the complete pathway of the duct

With 3D-bSSFP alone, we clearly tracked the thoracic duct and identified areas of leakage in some severe cases where 3D-bSSFP complemented T2w for confirmation of leakage sites. Improved tracking in 3D-bSSFP was likely due to higher SNR and cardiac-triggered acquisition along with dual-phase 3D-bSFFP whole-heart imaging [30, 31], an innate strength of this sequence (Fig. 3 and Additional files 2, 3, 4 and 5). However, image acquisition with either single or dual phase did not impact lymphatic visualization.

Most patients had type 2 lymphatic malformation (45%). We compared and applied the T2w-based classification system to 3D-bSSSFP and found good visual correlation in both modalities, thus improving our understanding and grading of lymphatic malformations (Additional files 6, 7, 8, 9, 10, 11, 12 and 13). Interestingly, we observed increased tortuosity of the thoracic duct, predominantly in types 2 and 3, and associated disruption of the thoracic duct in type 4. This tortuosity is consistent with a recent study that showed a higher tortuosity index of the thoracic duct in Fontan patients than in healthy controls [15]. These authors examined Fontan patients ≥ 18 year old, whereas we observed tortuosity even in patients who underwent bidirectional Glenn anastomosis, indicating the disruption of lymphatic morphology begins as early as 1–2 year of age. On rare occasions, tortuosity may be observed in utero in single ventricle patients [32].

**Fig. 4:**
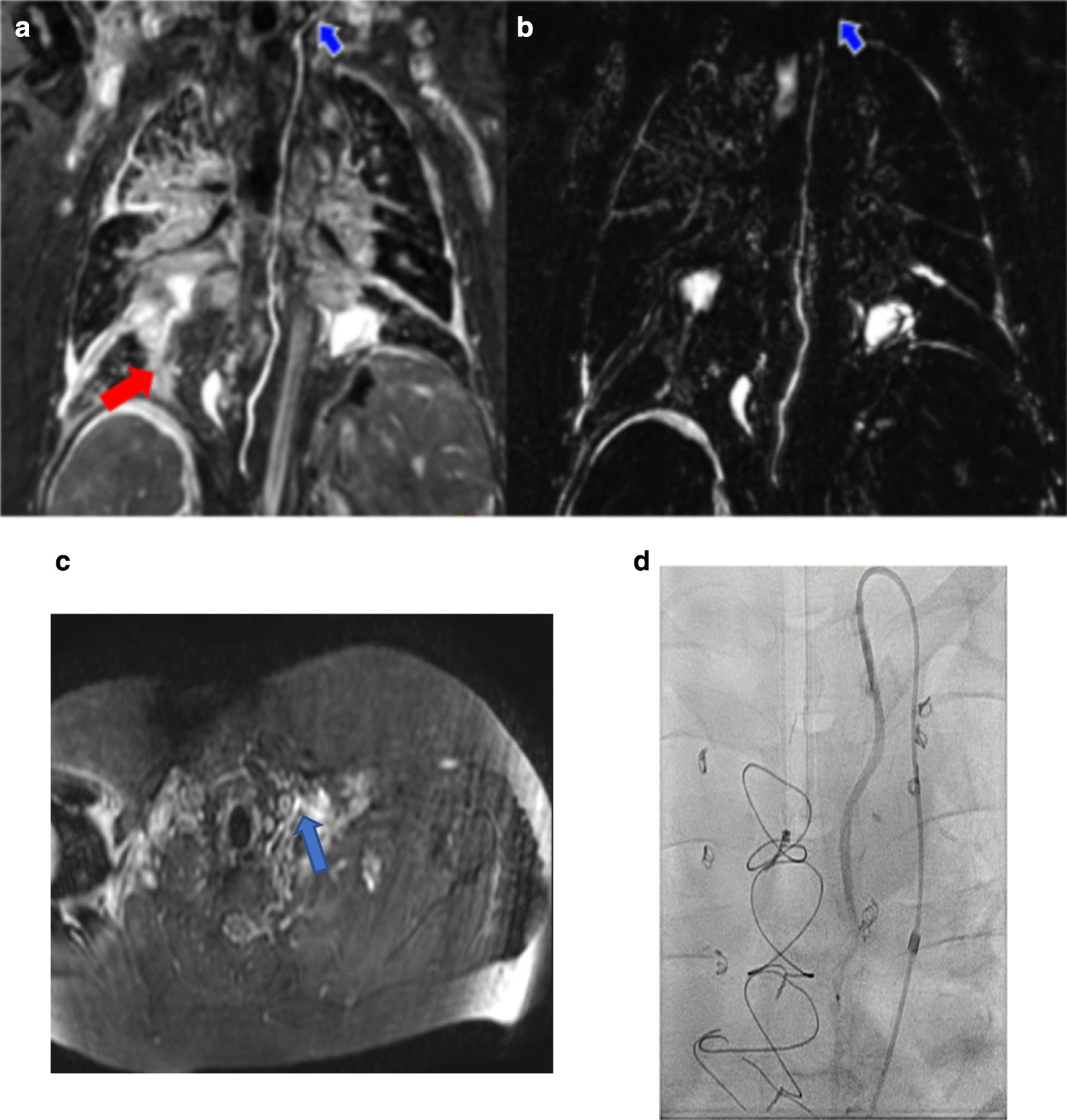
**a** A curved planar reformat of a 3D-bSSFP image. The course of the thoracic duct and lymphatic leakage in the thorax (red arrow) are shown. **b** A T2w image with loss of lymphatic duct visualization around the left lung apex (blue arrow). **c** 3D-bSSFP image in the axial upper thorax. Thoracic duct traversing from posterior to anterior and eventually draining into the internal jugular vein (blue arrow). **d** 3D-bSSFP guidance used for quick fluoroscopic-guided retrograde thoracic duct access, leading to a quick, successful lymphatic occlusion procedure in the catheterization lab with low radiation exposure

Our ultimate goal regardless of the sequence used was to improve diagnostic imaging and eventually guide successful lymphatic interventions. We performed DCMRL during invasive CMR single ventricle evaluation in a few cases, which helped us track the lymphatic pathway during contrast injection. DCMRL followed by 3D-bSSFP helped us identify and confirm lymphatic anatomy as well as understand its relationship with surrounding vascular structures including collaterals. However, due to the invasive nature of DCMRL and associated challenges with lymph node access, DCMRL is performed in few centers worldwide. In our institutional experience, prior to lymphatic interventional procedures, overlaying segmented 3D-bSSFP along with T2w and DCMRL data over fluoroscopy in the catheterization lab helped us gain retrograde access into the lymphatic system in most cases (Fig. 4). We also achieved good lymphatic interventional outcomes, especially in heterotaxy and other complex single ventricle patients. DCMRL provides critical dynamic information regarding the presence and patency of the thoracic duct and other accessory lymphatic channels, but information is sometimes inadequate due to technical issues or lack of infrastructure to perform a thorough DCMRL study. In such situations, complementary non-invasive 3D whole-heart bSSFP and T2w imaging provide superior anatomic details that can help interventionalists plan their approach for lymphatic interventions.

## Conclusions

3D whole-heart bSSFP datasets provide a complementary view of the lymphatic system to T2w lymphangiography for complete visualization and tracking of the thoracic duct from cisterna chyli to its drainage site and for the assessment of lymphatic malformations. Since 3D-bSSFP images are often acquired in patients requiring evaluation of the lymphatic system, complete visualization of the thoracic duct can be gained with review of existing datasets and no additional scan time. In cases of suboptimal T2w lymphangiography imaging, utilizing 3D-bSSFP for complete evaluation of the thoracic duct reduces the need for a complex, prolonged DCMRL procedure. Thus, this approach in addition to overlaying acquired data reduces the overall time for interventional access and radiation exposure, promoting successful catheterization-based lymphatic intervention in complex congenital cardiac diseases.

## Supplementary Information

**Additional file 1.** VF_1_T2 incomplete track—T2w shows tracking of thoracic duct from cisterna chyli to the midthorax where there is loss of signal below the left main bronchus consistent with incomplete tracking of the thoracic duct.

**Additional file 2.** VF_1_SSFP incomplete track—3D-bSSFP of the same patient shows tracking of the thoracic duct from cisterna chyli to its final draining site. Note at the level of mid-thorax, it crosses right of midline eventually draining into right supraclavicular plexus (normal variant).

**Additional file 3.** VF_2_T2 retrocardiac signal loss—T2w shows poor signal or near loss of signal from thoracic duct in the mid-thorax just below carina which may be secondary to cardiac motion. Also note, there is increased perihilar T2 signal from lymphatic leak and likely peri-Fontan conduit lymphatic channels.

**Additional file 4.** VF_2_SSFP retrocardiac signal loss—3D-bSSFP (dual phase) of the same patient shows complete tracking of thoracic duct from cisterna chyli to mid-thorax behind and below the carina where it divides and ascends cranially and drains into left innominate vein and left internal jugular vein junction and the right branch drains into right supraclavicular plexus.

**Additional file 5.** VF_3_G1 T2—Grade-1 lymphatic abnormality with T2w imaging signal showing minimal amount of abnormal lymphatic channels in the mediastinum or neck.

**Additional file 6.** VF_3_G1 SSFP—Corresponding grade-1 lymphatic abnormality as shown by 3D-bSSFP with minimal abnormal lymphatic channels

**Additional file 7.** VF_4_G2 T2—Grade-2 lymphatic abnormality with T2w imaging showing increased signal intensity within bilateral supraclavicular regions without significant extension into the mediastinum.

**Additional file 8.** VF_4_G2 SSFP—Corresponding grade-2 lymphatic abnormality as shown by 3D-bSSFP with increased abnormal lymphatic channels seen in the bilateral supraclavicular regions without significant extension into the mediastinum.

**Additional file 9.** VF_5_G3 T2—Grade-3 lymphatic abnormality with increased T2w signals showing extension of abnormal lymphatic channels into the mediastinum. Note abnormal peri-Fontan lymphatics and increased tortuosity of the thoracic duct.

**Additional file 10.** VF_5_G3 SSFP—Corresponding grade-3 lymphatic abnormality as shown by 3D-bSSFP with abnormal lymphatics and associated tortuosity of thoracic duct.

**Additional file 11.** VF_6_G4 T2—Grade 4 severe lymphatic abnormality with extensive T2w signals and disruption of thoracic duct with involvement of supraclavicular, mediastinum and interstitial region of lung parenchyma with evidence of chylothorax.

**Additional file 12.** VF_6_G4 SSFP—Corresponding grade-4 lymphatic abnormality with abnormal hyperintense signal in the mediastinum, interstitium of lung and pleural cavity consistent with severe form of lymphatic abnormality.

**Additional file 13.** VF_7_SSFP_T2w overlay—Overlay of 2 co-registered coronal 3D-bSSFP and T2w scans (left panel) showing improved visual tracking of the thoracic duct. Corresponding T2w shown in the right panel. Further description in Figure 2.

## Data Availability

The datasets used and/or analyzed during the current study are available from the corresponding author on reasonable request.

## Abbreviations

bSSFP: Balanced steady-state free precession
CMR: Cardiovascular magnetic resonance
DCMRL: Dynamic Contrast Enhanced Cardiovascular Magnetic Resonance Lymphangiography
DICOM: Digital Imaging and Communications in Medicine
FOV: Field of view
IQR: Interquartile range
IRB: Institutional Review Board
MIP: Maximum intensity projection
PACS: Picture Archiving and Communication System
SNR: Signal-to-noise ratio
T2w: T2-weighted
TE: Echo time
TR: Repetition time
TSE: Turbo spin echo
